# Research and Development of Electrostatic Accelerometers for Space Science Missions at HUST

**DOI:** 10.3390/s17091943

**Published:** 2017-08-23

**Authors:** Yanzheng Bai, Zhuxi Li, Ming Hu, Li Liu, Shaobo Qu, Dingyin Tan, Haibo Tu, Shuchao Wu, Hang Yin, Hongyin Li, Zebing Zhou

**Affiliations:** 1 MOE Key Laboratory of Fundamental Physical Quantities Measurement, Hubei Key Laboratory of Gravitation and Quantum Physics, School of Physics, Huazhong University of Science and Technology, Wuhan 430074, China; abai@mail.hust.edu.cn (Y.B.); lizhuxi@hust.edu.cn (Z.L.); liuli157@hust.edu.cn (L.L.); qushaobo@hust.edu.cn (S.Q.); tandy@hust.edu.cn (D.T.); scwu@hust.edu.cn (S.W.); yinhangcge@hust.edu.cn (H.Y.); hongyin83li@hust.edu.cn (H.L.); 2 Institute of Geodesy and Geophysics, Chinese Academy of Sciences, Wuhan 430077, China; huming@whigg.ac.cn (M.H.); tuhaibo@whigg.ac.cn (H.T.)

**Keywords:** electrostatic accelerometer, inertial sensor, satellite gravity recovery, gravitational wave detection in space

## Abstract

High-precision electrostatic accelerometers have achieved remarkable success in satellite Earth gravity field recovery missions. Ultralow-noise inertial sensors play important roles in space gravitational wave detection missions such as the Laser Interferometer Space Antenna (LISA) mission, and key technologies have been verified in the LISA Pathfinder mission. Meanwhile, at Huazhong University of Science and Technology (HUST, China), a space accelerometer and inertial sensor based on capacitive sensors and the electrostatic control technique have also been studied and developed independently for more than 16 years. In this paper, we review the operational principle, application, and requirements of the electrostatic accelerometer and inertial sensor in different space missions. The development and progress of a space electrostatic accelerometer at HUST, including ground investigation and space verification are presented.

## 1. Introduction

The history of high precision space accelerometers with small measurement range but high resolution dates back to the 1950s, when they were originally used to monitor the motion of satellites for the purpose of satellite control [1] in space environment investigation missions. In the 1960s, the technology of satellite control followed a geodesic line in space with the guidance of a space inertial sensor originally proposed at Stanford University [2]. The first drag-free satellite, TRIAD, with a target drag-free level of 10^−1^ m/s^2^ was launched in 1972, and its most important experimental payload was an inertial sensor adopting an electrostatic scheme designated DISturbance COmpensation System (DISCOS) [3,4]. In the early 1970s, an electrostatic accelerometer designated Capteur Accelerometrique Triaxial Ultra Sensible (CACTUS), with a designed resolution of 10^−9^ m/s^2^, was developed by the Office National d’Etudes et de Recherches Aérospatiales (ONERA, France) and was used to test satellite focusing in the study of drag forces, such as residual gas and solar radiation pressure [5,6].

In early applications of the electrostatic accelerometer in space, a spherical proof mass (PM) was selected for its advantage of not requiring rotational control, so that servo control could be easily realized. However, for the electrostatic accelerometer with a spherical PM, the accuracy and linearity is very poor, the cross-talk between different axes is very significant, and it cannot be used to monitor the rotational motions of a satellite. Because of these disadvantages, ONERA began to consider cubic PMs in the development of accelerometers. A typical example is the electrostatic accelerometer Accelerometre Spatial Triaxial Electrostatique (ASTRE) applied to monitor the motions of a spacecraft [7]. Based on the successful development and application of ASTRE, some similar accelerometers with minor variations or adjustments for different missions were developed, such as the STAR, SuperSTAR, and GRADIO accelerometers developed and employed for different satellite Earth field recovery missions, the CHAllenging Minisatellite Payload (CHAMP), Gravity Recovery And Climate Experiment (GRACE), and Gravity field and steady-state Ocean Circulation Explorer (GOCE) missions, respectively [8]. In the United States, some other electrostatic accelerometers have also been developed separately, such as the Miniature Electrostatic Accelerometer (MESA), the purpose of which was to monitor the motions of the Space Shuttle along three translational axes. The MESA was developed by Bell Aerosystems, who selected a thin-walled cylinder with a thin central flange to be the proof mass [9]. In the Swarm mission, which was launched on 22 November 2013 to study the Earth’s magnetic field, several onboard accelerometers were applied for observing the non-gravitational accelerations derived from the thermosphere’s density and wind [10]. Working in a different mode, the electrostatic accelerometer can also be used as a geodesic reference and be nominally called an inertial sensor in space gravitational wave detection missions such as LISA [11]. In order to verify the key technologies affecting the performance of the inertial sensor, a technology demonstration mission designated LISA Pathfinder was launched on 3 December 2015. The results of LISA Pathfinder indicate that the performance of the inertial sensor has already achieved the requirement of LISA [12]. An electrostatic accelerometer is also quite useful in fundamental physics, such as the MICROSatellite pour l’Observation du Principe d’Equivalence (MICROSCOPE) mission, which is aimed at testing the weak equivalence principle (WEP) in space down to an accuracy of 10^−15^. MICROSCOPE was proposed by ONERA and the Centre d’Etudes et de Recherches en Geodynamique et astrometrie (CERGA, France) and was launched on 25 April 2016. The electrostatic accelerometer is the most important payload for detecting the weak force due to the possible deviation of WEP in this mission [13].

Since 2000, a space electrostatic accelerometer has been under development at Huazhong University of Science and Technology (HUST), China, in order to facilitate space missions such as Test of the Inverse-Square law in Space (TISS) [14], Test of Equivalence Principle in space with Optical readout (TEPO) [15], space gravitational wave detection (TianQin) [16], and native satellite gravity measurement in China [17]. In this review, the basic principle of the accelerometer and the typical applications of a space accelerometer for different space missions are introduced and discussed. The development details and progress of the development of the electrostatic accelerometer at HUST are then presented. Specifically: (1) the low-noise capacitive displacement sensor and electrostatic actuators that meet the requirement of the above-mentioned missions have achieved; (2) the high-voltage levitation and fiber suspension facilities to investigate the function and performances of electrostatic accelerometers on the ground have been developed and constructed; and (3) the experimental results of a flight model tested in orbit for more than three years have been analyzed.

## 2. Principle of the Accelerometer

An accelerometer consists of at least a PM and a frame surrounding the PM, and its function is based on Newton’s Second Law. As the PM and the frame are subjected to different forces, the relative displacement between them will vary with time.

**Figure 1:**
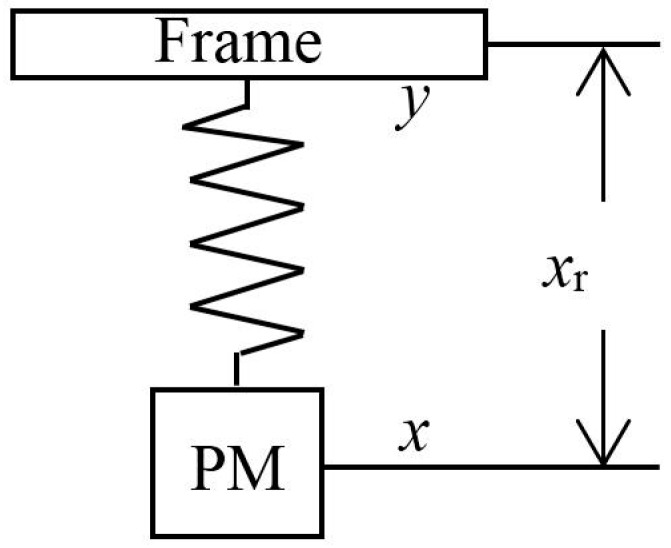
Schematic of spring accelerometer.

For an open-loop mechanical accelerometer with a spring linkage, as illustrated in Figure 1, the PM is isolated from external forces in ideal conditions, the relative displacement is proportional to the acceleration of the frame with respect to the local inertial reference frame, and its sensitivity is inversely proportional to the square of the natural frequency of the spring-mass oscillator system at very low frequencies. In Figure 1, *x* and *y* represent the displacement of the PM and the frame with respect to the local inertial reference frame, respectively, and *x*_r_ = *x* − *y* represents the relative displacement between the PM and the frame.

Neglecting the damping of the spring oscillator, the equation of motion of the PM is given by:

(1) $${\overset{‥}{x}}_{r}+\omega_{0}^{2}X_{r}=-\overset{‥}{y}$$

where *ω*_0_ is the natural angular frequency of the spring-mass oscillator.

From Equation (1), we obtain that the acceleration of the frame at low frequencies (*ω* << *ω*_0_) is dependent on the length of the spring, *x*_r_:

(2) $$a\approx \overset{‥}{y}=-x_{r}\omega_{0}^{2}$$

It is obvious that there are two ways to improve the intrinsic detection capability of this type of accelerometer: the first is to improve the resolution of the position transducer, and the second is to decrease the natural frequency of the oscillator.

Therefore, the mechanical spring linkage is usually replaced by softer and low-dissipation suspension schemes. Thus, high-precision accelerometers with pico-*g* or even better resolution based on an electrostatic control scheme [18] and the superconductive magnetic suspension scheme have achieved [19]. In addition, some other schemes, such as optical and atomic techniques, are being considered [20]. The softer linkage cannot only improve its sensitivity, but also suppress the back-action effect due to relative motion fluctuation or displacement sensing noise. However, the PM or the frame, in general, must be servo-controlled motionlessly with respect to each other for supersoft-linking-type accelerometers.

**Figure 2:**
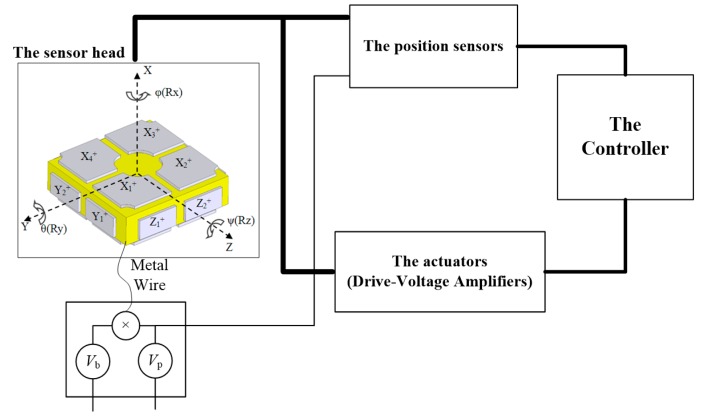
Schematic of HUST electrostatic accelerometer.

A schematic of the HUST closed-loop electrostatic accelerometer is shown in Figure 2. It consists of a sensor head, a displacement transducer, a controller, and an actuator. The sensor head consists of a PM and the surrounding electrode cage, where the six-degree-of-freedom (DoF) motions of the PM with respect to the electrode cage are measured by a six-channel capacitive displacement transducer, and then the low-frequency feedback voltages calculated from the controller are applied on the electrodes by drive-voltage amplifiers. Finally, the PM is controlled motionlessly with respect to the cage. In this case, the feedback voltage could indicate the differential forces acting on the PM and satellite, so that we can obtain the gravitational gradient or the non-gravitational forces acting on the satellite, such as air drag and light pressure. Here, a direct DC bias voltage, *V*_b_, and a high-frequency (i.e., 50–100 kHz) pumping voltage, *V*_p_, are applied to the PM by a soft metal wire, which help to linearize the electrostatic actuator and drive the capacitance transform bridge, respectively.

The measurement of electrostatic accelerometer can be expressed as:

(3) $$a_{\mathrm{elec}}=a_{\mathrm{sc}}+a_{n,\mathrm{acc}}$$

where *a*_elec_ means the acceleration acting on the PM by the electrostatic suspension, and it can be calculated by multiplication of the measurement feedback voltage *V*_fed_ and the calibrated transfer function of the electrostatic actuator *H*_a_. *a*_sc_ represents the acceleration of the spacecraft. *a*_n,acc_ is the residual acceleration disturbances and it could be given by:

(4) $$a_{n,\mathrm{acc}}=a_{\mathrm{ext},n}+a_{\mathrm{thermal}}+(\omega^{2}+\omega_{e}^{2})x_{n}+H_{a}V_{\mathrm{out},n}$$

where *a*_ext,n_ is the external disturbances acting on the PM which are induced by space and spacecraft environment. *a*_thermal_ here is the damping effect of the metal wire which is linked to the PM. *x*_n_, and *V*_out,n_ represent the displacement noise and readout voltage noise, respectively; *ω* is the natural angular frequency and *ω*_e_, represent the parasitic angular frequency associated to a negative stiffness because of the back-action of the capacitive transducer, which is mainly dependent on the bias voltage *V*_b_, capacitance *C*_0_, gap *d*_0_ and the mass of the PM.

This indicates that the electrostatic accelerometer must work in a closed-loop situation. The intrinsic noise of the electrostatic accelerometer itself is mainly limited by the displacement and readout voltage noises. But in actual applications, the external disturbances must be considered such as the electromagnetic and thermal environmental noises, the coupling influence from the spacecraft, and so on.

When the PM is servo-controlled to follow the spacecraft, this is called acceleration measurement mode. It has been successfully used in the CHAMP, GRACE and GOCE missions, but when the PM operates close to the free-falling state and acts as the geodesic reference; the spacecraft can then follow the PM by employing the thruster drag-free control system [3]. We call this scheme the geodesic reference mode, which is usually adopted in high-precision gravitational experiments in space to improve the microgravity level of a spacecraft. In this mode, the non-gravitational forces acting on the spacecraft such as the atmospheric drag, the solar radiation and so on will be compensated by the thrusters, namely the spacecraft will follow with the PM and the residual acceleration disturbances of the PM *a*_n,gr_ can be given by:

(5) $$a_{n,\mathrm{gr}}=a_{\mathrm{ext},n}+(\omega^{2}+\omega_{e}^{2})(x_{n}+x_{\mathrm{df},n})$$

where *x*_df,n_ represents the control level of the spacecraft by the drag-free control, which influenced by the drag-free external control forces acting on the spacecraft and the control loop gain. This operation mode is mainly suitable for the space gravitational wave detection missions such as LISA. In LPF mission, the relative acceleration noise between two free-falling reference proof masses on one satellite are measured [12].

## 3. Typical Applications of Space Electrostatic Accelerometer

### 3.1. Application in the Measurement of the Earth's Gravity Field

In acceleration measurement mode, the largest successful application of space electrostatic accelerometers is in the recovery of global Earth gravity field missions. The accelerometer, as a force probe, is used to measure the non-gravitational force acting on the satellite. For example, in order to recover the parameters of the global Earth’s field, the Global Navigation Satellite System (GNSS, including GPS and BeiDou) and inter-satellite ranging(using microwave or laser measurement) are used to measure the position and time variation of the trajectories of the satellites, which aim at deducing the total force acting on the satellites. In high-precision accelerometers, such as STAR in the CHAMP mission and SuperSTAR in the GRACE mission, the non-gravitational forces acting on the satellites are measured simultaneously. The differential between the total force and the non-gravitational force is the gravitational force, described by the parameters of the global gravity field.

The German CHAMP mission, launched in July 2000 at an initial altitude of 454 km, measured the global magnetic and gravity fields and the Earth’s atmosphere until September 2010. The three-axis STAR accelerometer is integrated at the center of mass of the satellite, presents a measurement range of ±10^−4^ m/s^2^ and exhibits a resolution of better than 3 × 10^−9^ m/s^2^/Hz^1/2^ for the highly sensitive axes within the measurement bandwidth from 0.1 mHz to 0.1 Hz [8]. The following GRACE mission, consisting of two identical satellites separated by approximately 220 km on the same quasicircular orbit, was launched on 17 March 2002 at an initial altitude of 500 km. Taking advantage of the CHAMP mission experience, the SuperSTAR accelerometer is similar to the STAR accelerometer, while its measurement noise level is 1 order of magnitude better, leading to a noise level of 1 × 10^−10^ m/s^2^/Hz^1/2^ along the highly sensitive axes with a reduced range of ±5 × 10^−5^ m/s^2^ [21,22]. The GRACE Follow-on Mission will use the same method to map gravitational fields; it is scheduled for launch in 2018. The two GRACE Follow-on satellites will use the same kind of microwave ranging system as GRACE, but they will simultaneously demonstrate laser ranging with approximately 20 times the resolution of microwave ranging. The accelerometer will realize a noise level better than 10^−10^ m/s^2^/Hz^1/2^, and special designs are being considered to improve the thermal characteristics of the accelerometer [23].

The GOCE mission is the first mission to use the concept of satellite gravity gradiometry in space to obtain higher harmonics of the Earth’s gravity mapping. The electrostatic gravity gradiometer (EGG) on the GOCE mission, constructed with three pairs of three-axis electrostatic accelerometers, was designed to measure the gradient components of the Earth’s gravity field. Each pair of accelerometers is identical, separated by approximately 0.5 m. The GOCE satellite was launched in March 2009, and the in-orbit data shows that the six accelerometers are fully operational as drag compensation sensors as well as serving as scientific instruments. After being calibrated carefully through a series of methods, the gradiometer reached a 10–20 mE/Hz^1/2^ accuracy at tens of mHz and an outstanding accelerometer in-orbit noise level of approximately (3–6) × 10^−12^ m/s^2^/Hz^1/2^ [24]. Even then, the major error sources come from the intrinsic noise of the electrostatic accelerometer and coupling from the satellite environment. Thus, in order to further improve the resolution, two schemes were designed by reducing the dynamic range and choosing a much heavier PM to suppress thermal noise limited by the discharging gold wire. A higher resolution, of approximately 7 × 10^−13^ m/s^2^/Hz^1/2^, could then be achievable in future satellite gradiometry missions [25].

Regarding the Gravity Recovery and Interior Laboratory (GRAIL) mission [26], the purpose of which is to map the Moon’s gravity field, the satellite gravity gradiometry method can also be considered. It can determine the Moon’s gravity field with a higher resolution and obtain the medium- and short-wavelength component information with greater accuracy. A model with a high accuracy of 14 mGal and a geoid with an accuracy of 20.5 cm can be realized with a gradiometer accuracy level of approximately 30 mE/Hz^1/2^ [27].

### 3.2. Application in Space Gravitational Wave Detection

In space gravitational wave detection missions, the inertial sensor works in “geodesic reference mode” and plays the role of a gravitational probe [28]. In order to detect gravitational waves, the PM of the inertial sensor acts as not only an object responding to the time-space variance induced by the passage of the gravitational waves but also as a free-falling reference with which to guide the control of the spacecraft with micro-Newton thrusters. According to the requirement of the LISA mission, the residual disturbance of the PM should be controlled below 3 × 10^−15^ m/s^2^/Hz^1/2^ in the measurement bandwidth from 0.1 mHz to 0.1 Hz [29]. The LISA mission is a joint European Space Agency/U.S. National Aeronautics and Space Administration (ESA/NASA) mission for detecting low-frequency gravitational waves in space, which have been studied since 1993 [30]. In response to the call of the ESA for L3 mission concepts, the LISA Mission consortium submitted the proposal for LISA on 13 January 2017. LISA consists of a triangular formation with three spacecraft in an Earth-trailing heliocentric orbit separated by 2.5 million km [31]. In each spacecraft, there are two inertial sensors with two PMs inside, which will provide the reference frame for the satellite and guide the drag-free control system to compensate for the non-gravitational force acting on the satellite using a thruster array. As gravitational waves pass the triangle, they will squeeze and stretch the space between the separations, and they can then be detected by delicate laser interferometers, which continuously monitor the tiny changes in the long separations at a level of tens of picometers.

LISA Pathfinder is a pioneer mission that was proposed in 1998 to test key technologies such as the inertial sensor, laser interferometer, micro-Newton thrusters, and drag-free control for the LISA mission [32]; it was launched on 3 December 2015. LISA Pathfinder carries two payloads, the European-provided LISA Technology Package (LTP) and the NASA-provided Disturbance Reduction System (DRS). In LISA Pathfinder, one laser arm is effectively reduced to approximately 38 cm inside a single spacecraft. The two cubic PMs both serve as mirrors for the laser interferometer, and one PM serves as an inertial reference for the drag-free control system of the spacecraft, which will be used for the LISA mission. The position and attitude of the PMs are controlled by a combination of the inertial sensors and spacecraft micro-thruster drag-free control. The in-orbit results show that the relative acceleration noise level is approximately 5 fm/s^2^/Hz^1/2^ between 0.7 and 20 mHz [12], which is better than the expectation of LISA Pathfinder. With the improvement of the vacuum of the PMs and temperature stabilization, much better results should be reported quickly.

**Figure 3:**
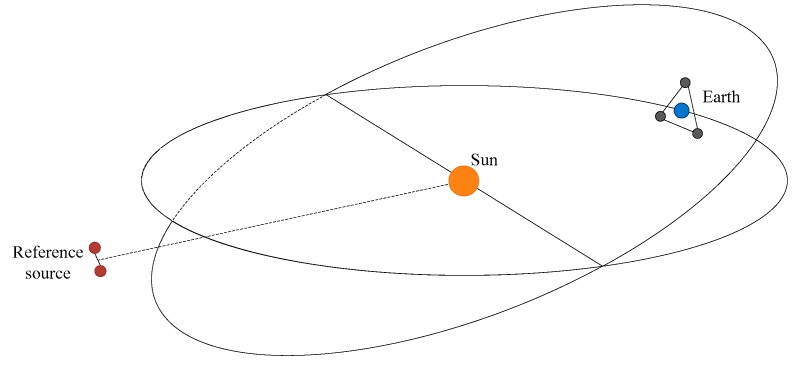
Illustration of preliminary concept of TianQin mission.

The TianQin mission is a new proposal for a spaceborne detector of gravitational waves in the mHz frequency range [16]. An illustration of the preliminary concept of the TianQin mission is shown in Figure 3, in which three identical spacecraft form a nearly equilateral triangle in geocentric orbits with a semi-major axis at the 10^5^ km level. Each of the spacecraft will be equipped with two free-falling PMs inside. The key technologies rely on two components: the laser interferometer and the disturbance reduction system. The primary mission goal is to detect gravitational waves with anticipated properties from a single well-understood and easily accessible reference source, such as the ultracompact binary white dwarf RX J0806.3 + 152. All the aspects of the experiment are optimized using properties of a tentative reference source, and the present results show that the requirement for the residual acceleration is 10^−15^ m/s^2^/Hz^1/2^ at approximately 6 mHz. Some detailed designs of the mission, such as the scheme for the inertial sensing system of the disturbance reduction system, are still incomplete and will be fully demonstrated and confirmed as development proceeds.

As a key payload in spaceborne gravitational wave detectors, an extremely high requirement has arisen for the inertial sensor, which is definitely beyond the experience accrued from any existing missions. To develop and demonstrate a high-precision inertial sensor, a few delicate torsion pendulums have been constructed and developed [33,34,35], and a number of noise sources have been carefully studied, such as cross-coupling [36], thermal noise [37], and surface potential difference [38]. The qualification of the inertial sensor for such a noise level depends on its operation in space. The Trento group has been engaged in developing the inertial sensor for spaceborne gravitational missions like LISA and LISA Pathfinder. Several highly sensitive torsion pendulums have been developed in order to estimate the upper limit of the noise and characterize noise sources experimentally in the laboratory [39], where a gold-coated PM was suspended by a tungsten or fused silica fiber with a torque sensitivity of approximately 1 fNm/Hz^1/2^ at mHz frequencies [37]. The PM is hollow in order to maximize the sensitivity of the torsion pendulum to the torque noise arising from the surface effects of the PM. With the above-described weak force measurement facility, a few interesting effects have been carefully investigated, such as the electrostatic stiffness and the dielectric dissipation in the conductive surface. A few possible upgrades of the torsion pendulum are under study, with the goal of trying to meet the verification demand for the advanced inertial sensor for the LISA mission in the laboratory in the near future. A torsion pendulum has been also developed to investigate residual disturbances of the PM, such as patch effects. A torsion pendulum has been built at the University of Washington (UW) to measure the surface-potential variations between two gold-coated surfaces, with a noise level of approximately 30 μV/Hz^1/2^ at frequencies above 0.1 mHz [40]. In the interest of realizing further improvements, the workers at UW have used an ultraviolet LED to demonstrate both charging and discharging of the pendulum.

In addition, a few other projects have been proposed or have achieved testing of the gravitational law and have searched for new interactions using the inertial techniques, including Relativity Mission Gravity Probe B (GP-B) [41], Astrodynamical Space Test of Relativity using Optical Devices (ASTROD) [42], and DECi-hertz Interferometer Gravitational wave Observatory (DECIGO) [43].

### 3.3. Application in the Test of the Equivalence Principle in Space

The equivalence principle (EP), as a basic hypothesis of general relativity, has been an attractive test object for experimental scientists since it was first put forward. To improve the testing level of EP using the space environment, the Satellite Test of the Equivalence Principle (STEP) mission was proposed in 1972 at Stanford University [44], following some other similar missions, including Galileo Galilei (GG), MiniSTEP, and QuickSTEP, proposed by different organizations [45]. MICROSCOPE was proposed in 1999 by ONERA and launched on 25 April 2016. It is the first mission specifically designed to test the EP in space at the 10^−15^ level, which is 2 orders of magnitude better than the current ground-based experiments, and which could allow us to rule out theories beyond general relativity that predict a WEP violation at approximately the level of 10^−15^, or to complete general relativity if a WEP violation is detected. In the MICROSCOPE mission, the motions of the two PMs, which have different compositions, i.e., Pt and Ti, are monitored by capacitive transducers and controlled to be motionless by electrostatic forces [46]. The feedback electrostatic forces applied to the two PMs implies differential accelerations along the sensitive axis corresponding to the violation of the EP. The designed resolution of the differential SAGE accelerometers in MICROSCOPE is expected to be approximately 2 × 10^−12^ m/s^2^/Hz^1/2^ in the frequency band of 0.1 mHz to 0.03 Hz [47].

**Figure 4:**
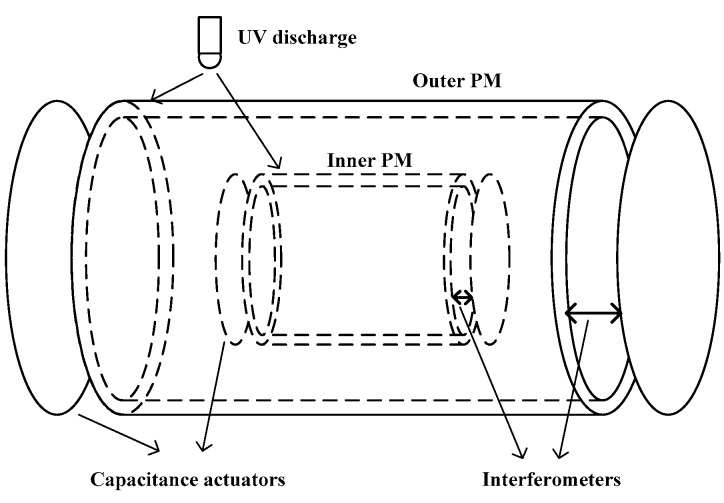
Schematic of PMs of TEPO.

The TEPO project was proposed to test the EP at the level of 10^−17^ for different composition bodies by HUST, in which the technologies used in the MICROSCOPE and LISA Pathfinder, such as the heterodyne laser interferometer, precision electrostatic accelerometers, and the ultraviolet (UV) charge management system, are expected to be adopted [15]. In the TEPO mission, the PMs are designed to be hollow concentric cylinders, the same seminal design as in the MICROSCOPE mission, with an outer titanium PM and an inner platinum PM. The relative motion of the two PMs in the sensitive axis, which are affected by the possible EP violation, are monitored by a laser heterodyne interferometer, and then controlled to be motionless by electrostatic actuators, as shown in Figure 4. Instead of the gold wires employed in the MICROSCOPE mission, a UV discharge system, as developed and tested with the GP-B [41] and LISA Pathfinder [12] proof masses, is used to discharge the PMs based on the photoemission effect, which can avoid the damping of the gold wires. Based on detailed analysis and theoretical calculations, the accuracy of the TEPO project based on the best level of the technologies mentioned above is estimated, and the results show that the resolution of the differential acceleration could reach 1.9 × 10^−1^^3^ m/s^2^/Hz^1/2^ at a frequency of 1 mHz; the EP could be tested at 8 × 10^−17^ with a 1-d integration.

### 3.4. Application in the Test of the Inverse-Square-Law in Space

**Figure 5:**
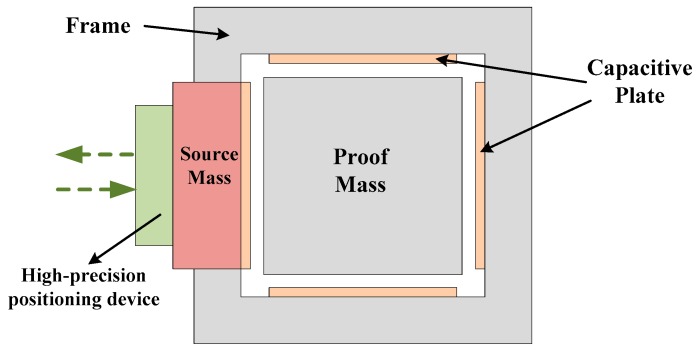
Schematic of TISS project.

The Test of the Inverse-Square-law in Space (TISS) project was proposed in 2006 to test the Newtonian gravitational law and to search for new interactions in a sub-millimeter range by an electrostatic accelerometer [14]. A schematic of the TISS project is shown in Figure 5. The proof mass is attached to the middle of the frame, and the source mass is fixed on a high-precision positioning device such as a piezoelectric transducer (PZT) platform. Six-degree-of-freedom capacitive sensors are used to detect the distance between the source mass and the PM, and then feedback voltages are applied on the capacitive plates, which makes the PM maintain its initial equilibrium position. The feedback voltages can represent the gravitational force of the PM attracted by the source mass. When the distance varies, the feedback voltage varies as well, and changes in the Newtonian gravity force can be calculated. The theoretical calculation showed that the strength factor α for the general Yukawa’s potential can be tested lower than the 10^5^ at the μm level range [14], when the resolution of the electrostatic accelerometer reaches 3 × 10^−10^ m/s^2^/Hz^1/2^ and the minimum distance between the source mass and PM can be periodically driven from 20 to 10 μm with a period of approximately 10–100 s. In this case, the result can be improved by a factor of 5–10 compared with current terrestrial experimental results.

## 4. Progress of Electrostatic Accelerometer Development at HUST

To advance space gravitational experiments such as the TISS and TEPO projects and the satellite Earth’s gravity recovery mission, our group at HUST began to study and develop high-precision space electrostatic accelerometers in 2000.

### 4.1. Sensor Head Manufacturing Technique

The sensor head of an electrostatic accelerometer usually consists of a PM and a surrounding electrode housing. The materials of the PM and housing electrodes are typically titanium and Ultra low expansion glass. The gap between the PM and the electrode housing affects the dynamic range, as well as the measurement resolution, of the accelerometer, and there is a tradeoff between these two parameters. The gap is generally chosen to be hundreds of micrometers, which requires an extremely high-precision manufacturing technique to fabricate the sensor head. The processing procedure mainly includes ultrasonic machining, wire-cutting machining, polishing, and coating, along with other processes. So far, our group has independently mastered all of the processing technology for building a sensor head, in which the accuracy of the flatness is better than 1 μm, and the perpendicularity is better than 5 arcsec.

### 4.2. Low-Noise Capacitive Transducer and Readout System

An important technology of accelerometer fabrication is development of an ultra-low noise electronics unit. Taking an accelerometer with a design resolution of 2 × 10^−12^ m/s^2^/Hz^1/2^ at the frequency range of 5 mHz to 0.1 Hz, for example, the capacitive transducer resolution should increase to 2 × 10^−7^ pF/Hz^1/2^ at 0.1 Hz, corresponding to approximately 4 pm/Hz^1/2^ for a 300-μm gap design, while the value of *ω_e_*^2^ is approximately 0.05 rad/s^2^, and the readout voltage noise should be controlled within 2 μV/Hz^1/2^ at 5 mHz. But in actual applications, the external disturbances must be considered such as the electromagnetic and thermal environmental noises, the coupling influence from the spacecraft, and so on.

**Figure 6:**
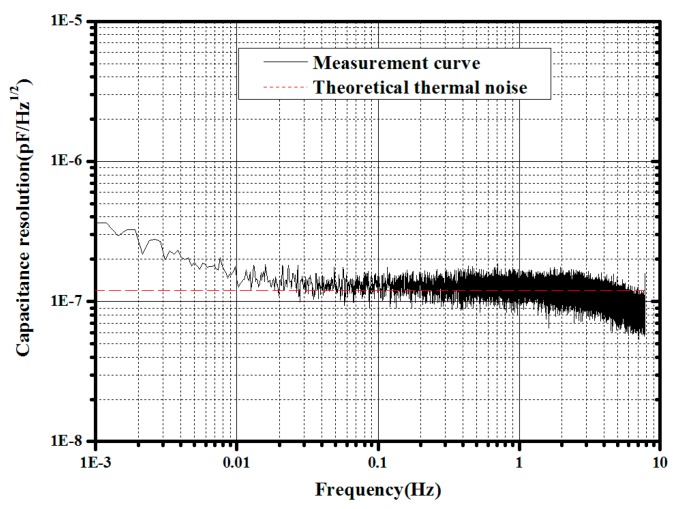
Noise power spectrum density of capacitive transducer.

A precise capacitive transducer based on a differential transform bridge and the Field-Programmable Gate Array (FPGA) technique has been carefully studied and developed [48], and currently, the noise level has increased to 1.6 × 10^−7^ pF/Hz^1/2^ down to 1 mHz [49], which is limited by the thermal noise of the front-end electronics as shown in Figure 6; this can satisfy the requirement of the displacement measurement for a 10^−12^ m/s^2^/Hz^1/2^-level accelerometer and is also suitable for position measurement of the PM in the TianQin project.

**Figure 7:**
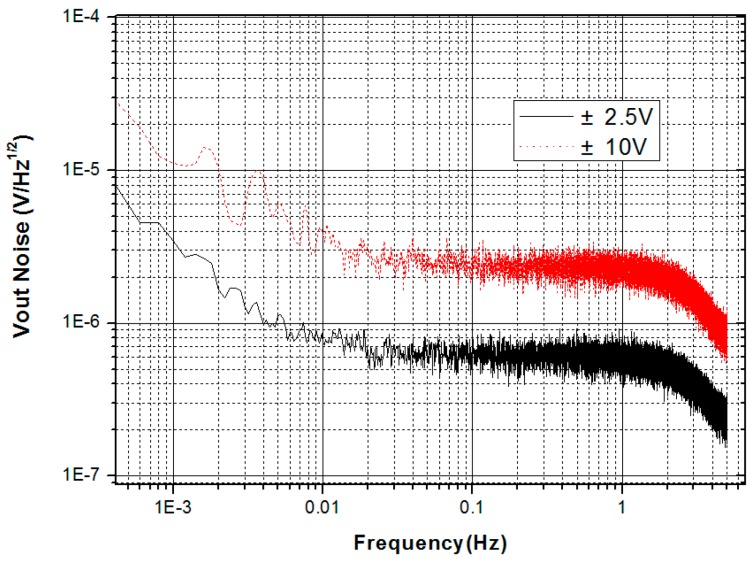
Noise power spectrum density of readout system.

In general, the output voltage noise for the electrostatic accelerometer is at a level of 10 μV/Hz^1/2^, which is limited by the quantum noise of a 16-bit digital-to-analog converter (DAC) and the thermal noise of a voltage-driven amplifier. To suppress the output noise, a direct voltage readout scheme is adopted using a high-precision analog-to-digital converter (ADC) (i.e., 20 bits or better) to measure the voltage applied on the control electrodes, which can suppress the electric and quantum noises of the DAC and voltage-driven amplifier due to the large open loop gain in the frequency band of interest. A voltage readout noise of approximately 2 μV/Hz^1/2^ was realized using this scheme [50]. Figure 7 shows that a noise level of approximately 0.6 μV/Hz^1/2^ was realized using a 24-bit ADC in the ±2.5 V range.

### 4.3. High-Voltage Levitation Test

For high-precision inertial sensors, accurate performance verification on the ground is mainly limited by Earth’s 1 g gravity acceleration. There are two ways to conquer this influence. One is to apply a high voltage on the upper electrodes to levitate the PM [18]; the other is to suspend the PM using a dedicated fiber. By using a high voltage to levitate the PM, ONERA has succeeded in testing the performance of a series of space accelerometers, such as the STAR, SuperSTAR, and GRADIO accelerometers. It should be noted that direct performance verification of these accelerometers on the ground using the high-voltage levitation scheme is limited at a level of 10^−8^ m/s^2^/Hz^1/2^ due to the residual seismic noise of the test bench and to the coupling from the strong vertical electrostatic field.

**Figure 8:**
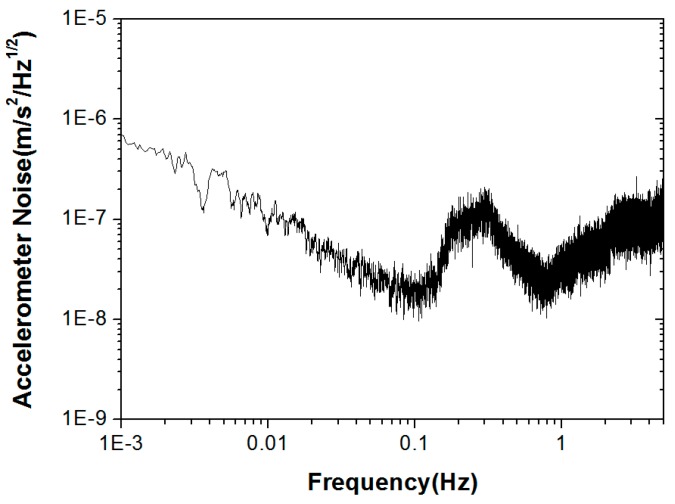
Acceleration measurement level using high-voltage-levitation scheme.

The high-levitation-voltage method is proposed to test various six-DOF control strategies, and it is also suitable for testing the engineering and flight models of accelerometers for space missions. At HUST, a titanium-alloy PM weighing approximately 70 g with a vertical gap of approximately 50 μm was levitated by a simple high-voltage actuator with an output range up to 900 V and a frequency bandwidth of 11 kHz, which is realized by an operational amplifier and a metal-oxide-semiconductor field-effect-transistor (MOSFET) combination [51]. The translation noise of the accelerometer increased to approximately 2 × 10^−8^ m/s^2^/Hz^1/2^ at 0.1 Hz, as shown in Figure 8, but is limited by seismic noise.

### 4.4. Fiber Suspension Test

**Figure 9:**
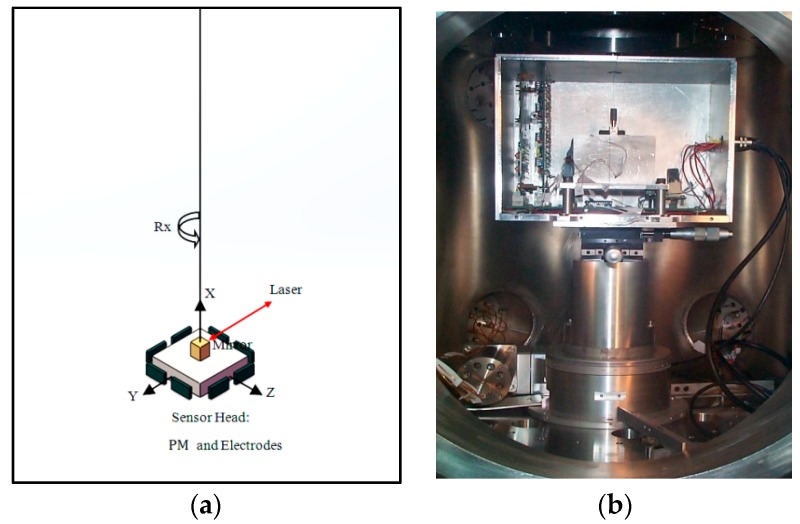
(**a**) Schematic of one-stage torsion pendulum; (**b**) experimental setup of torsion pendulum.

In addition to the high-voltage-levitation scheme, another scheme, namely the fiber suspension scheme, has been studied for a long period of time at HUST. A few electrostatically-controlled torsion pendulums, including one- and two-stage torsion pendulums, have been constructed to investigate electrostatically-controlled performance [34,36,53,54]. A one-stage electrostatically controlled torsion pendulum is a simple way to test an accelerometer with three DOFs; that is, two horizontal DOFs and a highly sensitive rotational DOF, which is shown in Figure 9.

**Figure 10:**
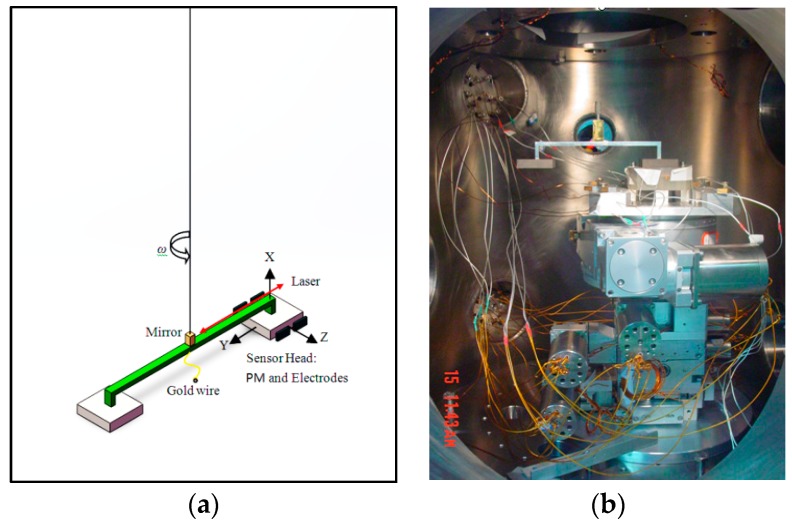
(**a**) Schematic of torsion balance; (**b**) experimental setup of torsion balance in vacuum chamber.

A one-stage electrostatically controlled torsion balance consisting of a PM and a counterweight connected to a balance bar, as shown in Figure 10, has a higher sensitivity along the horizontal direction. It is sensitive to the force actuated on the entire PM. Because of good suppression of seismic noise coupling along the sensitive axis in closed-loop control, it has been engaged to investigate the highly sensitive translational axis of the accelerometer.

In the electrostatically-controlled torsion pendulum scheme, the parasitic negative stiffness *k*_e_ induced by the capacitive transducer is much larger than that of the fiber, *k*_f_, since the capacitive gaps are usually quite small (much less than 1 mm). In this case, the torsion pendulum is unstable and cannot work in the absence of the servo control. Therefore, this scheme can be used to simulate the closed-loop operation of the accelerometer in flight. From this point of view, the scheme is slightly different from that used by the Trento group, who aim to investigate the residual disturbances of the PM using a free-torsion pendulum, where *k*_e_ is usually much smaller than *k*_f_ and the torsion pendulum is self-stable even without control (the capacitive gaps are designed to be much larger than 1 mm). Another advantage of this scheme is that it is helpful to suppress the influence of the seismic noise between the fiber suspension and electrode frame at low frequency due to its common mode rejection advantage [55]. Based on the applied forces necessary to keep a suspended PM centered in translation, the force noise of translationally-free torsion pendulums due to coupling to translational ground motion such as those used in Ref [37,38] is suppressed. However, our measurements allow a more representative test of the closed-loop accelerometer operation that is needed in many experiments.

**Figure 11:**
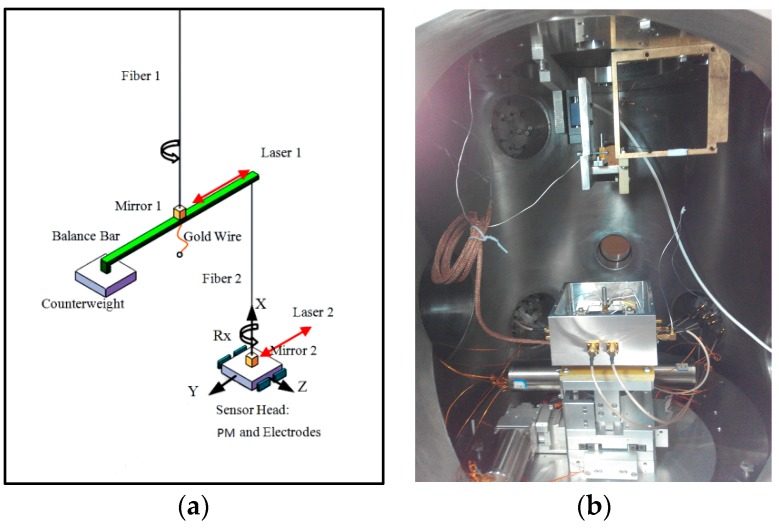
(**a**) Schematic of two-stage electrostatically-controlled torsion pendulum; (**b**) experimental setup of two-stage torsion pendulum in vacuum chamber.

**Figure 12:**
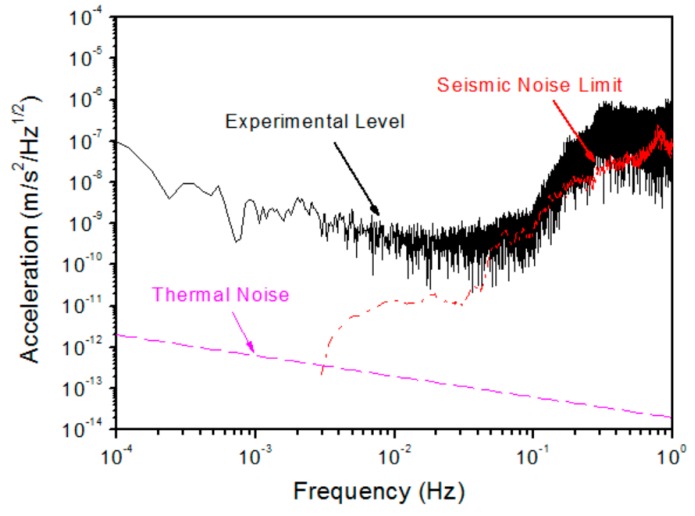
Acceleration measurement level based on two-stage pendulum.

An advanced torsion balance combining the advantages of a torsion pendulum and torsion balance, called a two-stage electrostatically-controlled torsion pendulum, has been developed to investigate the performance of high-precision accelerometers. This balance allows us to test the performance of both the translational and the rotational DOFs of the inertial sensor simultaneously and also helps to investigate the cross-talk effect between DOFs on the ground, which is considered to be one of the more challenging verifications. A schematic of the two-stage electrostatically controlled torsion pendulum and actual experimental setup in a vacuum chamber is shown in Figure 11. With this fiber suspension scheme, a noise level of 10^−10^ m/s^2^/Hz^1/2^ has been directly verified for the accelerometer, as shown in Figure 12.

To further suppress the effect of the seismic noise in testing the performance of an accelerometer or inertial sensor on the ground with a two-stage electrostatically controlled torsion pendulum, we have proposed a possible way that the capacitive electrode cage can be suspended by another pendulum. Theoretical analysis shows that the effects of the seismic noise can be further suppressed more than 1 order using the proposed approach [56]. By suspending the electrode cage, the preliminary experiment has verified that seismic noise coupling has been further suppressed by roughly 1 order, with a performance test noise level of 2 × 10^−11^ m/s^2^/Hz^1/2^.

**Figure 13:**
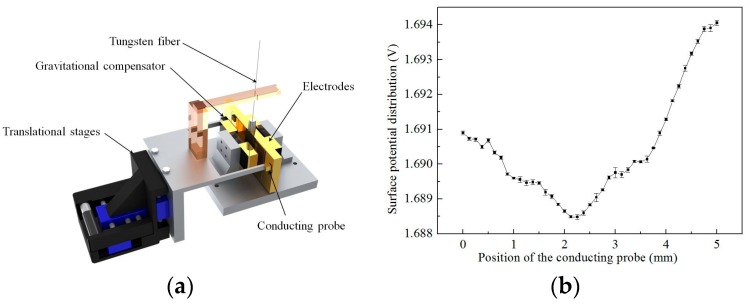
(**a**) Schematic of electrostatically-controlled torsion pendulum; (**b**) spatial variation of surface potential on proof mass measured by electrostatically-controlled torsion pendulum.

Meanwhile, we have also presented an electrostatically-controlled torsion pendulum with a scanning conducting probe to measure the charge distribution and its variation with better precision and higher resolution. The schematic of this novel scheme is shown in Figure 13a; this scheme combines the scanning capability of the Kelvin probe and the high precision of the torsion pendulum. Temporal variation of the surface potential can be measured at a level of 15 μV/Hz^1/2^ at 0.03 Hz, and the surface-potential distribution can be obtained at a level of 330 μV at a 0.125-mm spatial resolution, as shown in Figure 13b [57].

A test bench with a low-frequency vibration-isolation system is currently being constructed [52] and is expected to help improve the on-the-ground noise verification capability by 1 or 2 orders in the near future.

### 4.5. In-Orbit Test

**Figure 14:**
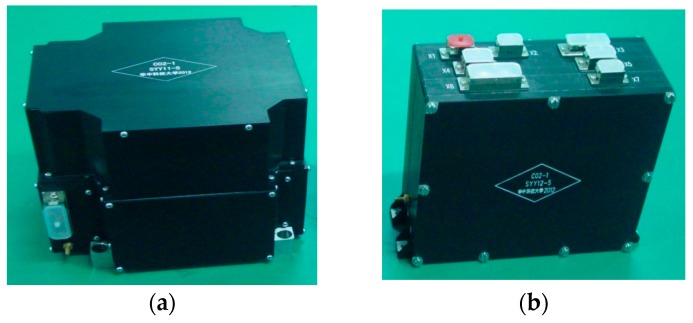
HSEA-I flight model: (**a**) sensor box and (**b**) electronics control box.

Although the accelerometer is tested on the ground by high-voltage suspension and torsion pendulum suspension, the ground working state is different from the space microgravity environment. Sufficient verification of the development technique of an accelerometer requires a spaceflight test. A flight model designated HSEA-I was developed at HUST, including a sensor box and an electronic control box, as shown in Figure 14. The accelerometer was launched aboard a technology experimental satellite in November 2013 and has been tested in orbit for more than three years to date [58].

The main objective of the HSEA-I flight experiment is to fully test the six-DOF control function of the electrostatic accelerometer over a long period of time in a space microgravity environment. The accelerometer was designed with a much higher measurement range to adapt to the anticipated microgravity level of the satellite. The tested intrinsic noise level on the ground is approximately 3 × 10^−8^ m/s^2^/Hz^1/2^ at approximately 0.1 Hz.

**Figure 15:**
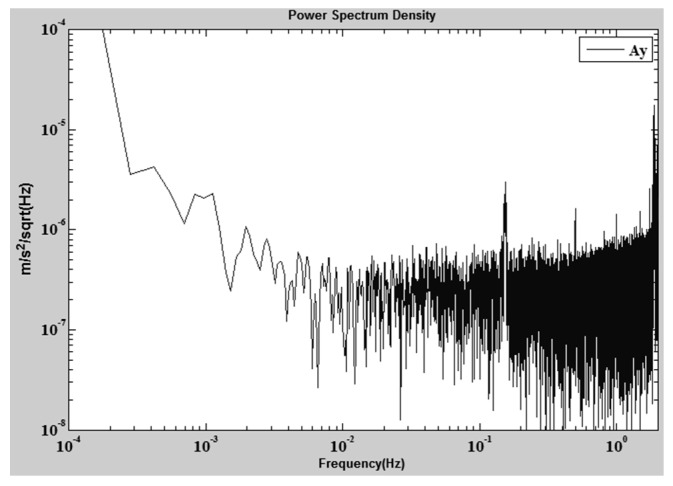
In-orbit noise spectrum of sensitive axis of HSEA-I.

The in-orbit data show that the six-DOF motions of the PM are always controlled within approximately 10 nm/Hz^1/2^ at 1 Hz. Meanwhile, we use the in-orbit data to estimate the relative distance between the center-of-mass (CoM) of the accelerometer and the satellite. The basic method is to compare the output of the accelerometer with the gyroscope data during attitude maneuvering, where the accelerometer is influenced mainly by the centrifugal acceleration and the linear acceleration induced from angular motion. A least square estimation method is used to estimate the three coordinate components of the CoM, and a position-estimation accuracy of approximately 6 mm is achieved [59]. During the in-orbit test, the noise level of the sensitive axis of the accelerometer, in the normal direction of the satellite’s orbital plane, is typically shown as in Figure 15. The noise level is approximately 1 order higher than the ground level. From a simulation analysis, the increase of the noise level is mainly the spectrum leakage effect from the satellite high-frequency accelerations due to the nonlinearity and the decimation aliasing effect of the accelerometer.

Until now, the accelerometer has worked almost with the same noise level in space. In addition, it has also successfully recorded the structural vibrations and attitude maneuvering of the satellite. A new bias calibration method has also been built, and the HSEA-I bias is being evaluated using the in-orbit data [58]. Based on the in-flight test results, the accelerometer development technology has been verified, and the overall performance of the space electrostatic accelerometer in orbit is even better than expected.

An improved high-precision electrostatic accelerometer was designed and developed for the TISS project beginning in 2014, and it is being tested in orbit on China’s first cargo spaceship, Tianzhou-1, which was launched on 20 April 2017. The electrostatic accelerometer has worked well in space until now, and we hope to make further studies of its performance in the future using long-term orbital data.

## 5. Discussion and Conclusions

In this paper, we have reviewed the electrostatic accelerometers developed by our group at HUST. The key technologies included the sensor-head manufacturing technique and realization of the high-precision capacitive position transducer and the low-noise readout circuits. The ground investigation and verification facility, including the high-voltage levitation system and several complicated torsion pendulum systems, have been set up. In particular, the performance of the 10^−10^ m/s^2^/Hz^1/2^ level electrostatic accelerometer can be directly validated based on the two-stage electrostatically-controlled torsion pendulum. Above all, two flight models of electrostatic accelerometers are being successfully tested in space to verify the entire system throughout its ongoing development.

Currently, we have designed a novel digital controller based on disturbance observation and rejection using the well-studied embedded model control (EMC) methodology [60], which will be tested experimentally. This method can also be used to study drag-free control for different space missions. In addition, several passive and active isolation benches are being studied; their purpose is to further suppress seismic noise in the high-voltage-levitation testing method. Meanwhile, an improved electrostatically-controlled torsion pendulum is being set up to measure the charge distribution and the variation of the PM with different materials. In order to investigate the magnetic bulk effects of the inertial sensor to be used in space gravitational wave detection missions, a massive PM combined with a pendulum system have been set up.

The above-mentioned progress and plans are important and promote Chinese space gravitational experiments, including the TISS, the EP, and space gravitational wave detection, among others. Moreover, the electrostatic accelerometer has been the key payload of several Chinese satellite gravity-measurement missions.
